# An Extended Kalman Filter for Magnetic Field SLAM Using Gaussian Process Regression

**DOI:** 10.3390/s22082833

**Published:** 2022-04-07

**Authors:** Frida Viset, Rudy Helmons, Manon Kok

**Affiliations:** 1 Delft Center for Systems and Control, Delft University of Technology, 2628 CD Delft, The Netherlands; m.kok-1@tudelft.nl; 2 Maritime and Transport Technology, Delft University of Technology, 2628 CD Delft, The Netherlands; r.l.j.helmons@tudelft.nl

**Keywords:** simultaneous localization and mapping, Kalman filtering, localization, magnetic field

## Abstract

We present a computationally efficient algorithm for using variations in the ambient magnetic field to compensate for position drift in integrated odometry measurements (dead-reckoning estimates) through simultaneous localization and mapping (SLAM). When the magnetic field map is represented with a reduced-rank Gaussian process (GP) using Laplace basis functions defined in a cubical domain, analytic expressions of the gradient of the learned magnetic field become available. An existing approach for magnetic field SLAM with reduced-rank GP regression uses a Rao-Blackwellized particle filter (RBPF). For each incoming measurement, training of the magnetic field map using an RBPF has a computational complexity per time step of $O(N_{p}N_{m}^{2})$, where $N_{p}$ is the number of particles, and $N_{m}$ is the number of basis functions used to approximate the Gaussian process. Contrary to the existing particle filter-based approach, we propose applying an extended Kalman filter based on the gradients of our learned magnetic field map for simultaneous localization and mapping. Our proposed algorithm only requires training a single map. It, therefore, has a computational complexity at each time step of $O(N_{m}^{2})$. We demonstrate the workings of the extended Kalman filter for magnetic field SLAM on an open-source data set from a foot-mounted sensor and magnetic field measurements collected onboard a model ship in an indoor pool. We observe that the drift compensating abilities of our algorithm are comparable to what has previously been demonstrated for magnetic field SLAM with an RBPF.

## 1. Introduction

Autonomous navigation using only onboard sensors is a desirable technology for various applications. There is no stable access to GNSS signals indoors, underground, or underwater, as they are blocked by building elements, earth and water, respectively [1,2,3]. In other environments, the use of GNSS signals for localization can also be challenging. Surface vehicles in harbours can be close to containers, bridges, or other industrial elements that can cause multi-path effects on the GNSS (Global navigation satellite system) measurements [4]. Autonomous navigation using only onboard sensors is challenging because of the drift in the position estimate obtained from sensor measurements that are independent of pre-deployed infrastructure [5]. Drift occurs when noisy measurements from, for example, gyroscopes, accelerometers, Doppler velocity logs or wheel encoders are integrated to estimate position without any absolute position measurements [6]. We will refer to the position estimates and orientation estimates obtained when integrating such noisy measurements as odometry. A range of other possible sensor readings may be available in these scenarios. The scope of our research is limited to the investigation of autonomous navigation using onboard odometry and magnetic field measurements.

Magnetic field simultaneous localization and mapping (SLAM) has been proposed to compensate for odometry drift when there is access to magnetic field measurements in a magnetic field with stationary spatial variations [7]. It has been demonstrated for indoor localization that magnetic field SLAM can be used to improve position estimates [8]. In environments with ferromagnetic structures, as for example in indoor environments, the magnetic field has rich spatial variation due to the magnetization of the metal [9]. Navigation using nonlinear variations in the ambient magnetic field has been proposed for a range of applications, such as indoor localization [1,8,10,11,12,13,14,15,16,17,18,19,20,21,22], underwater localization [23,24,25,26,27,28,29,30,31], and surface and aerial navigation [32,33]. Although [32] uses an extended Kalman filter (EKF) for localization in a learned magnetic field, the majority use a particle filter [1,10,11,12,13,15,16,17,18,22,25,27]. A comparative study has demonstrated that the particle filter is more accurate for underwater geomagnetic navigation in the case where the initial position is not known, while the EKF is more computationally efficient [34].

Computationally tractable magnetic field SLAM in three dimensions was proposed in [8], using a Rao-Blackwellized particle filter (RBPF) to simultaneously estimate the position and orientation of a pedestrian as well as the ambient magnetic field. A set of $N_{p}$ particles are used to represent the position and orientation [8]. The RBPF for magnetic field SLAM proposed by [8] uses Gaussian process (GP) regression to combine knowledge about the nature of the magnetic field from Maxwell’s equations with measurements of the magnetic field to create a magnetic field map. To this end, they build the magnetic field map for each particle using reduced-rank Gaussian process regression, which represents the magnetic field map as a linear combination of $N_{m}$ Laplace basis functions on hexagonal domains, and which represents the magnetic field map uncertainty as a matrix with $N_{m}^{2}$ entries. As each magnetic field map is represented with the weights of $N_{m}$ basis functions and the corresponding covariance of these weights, all of which require updating at each time step, the computational cost of updating the magnetic field map is $O(N_{p}N_{m}^{2})$ [8]. In the case where the particle filter is run on a parallelized architecture, such as FPGAs, the computation time dependence on the number of particles can be reduced dramatically [35,36]. The scope of our research is limited to improving the speed of Magnetic field SLAM on non-parallelized architectures. Magnetic field SLAM has also been demonstrated feasible using an RBPF with Laplace basis functions defined on a single, cubic domain [22].

**Figure 1:**
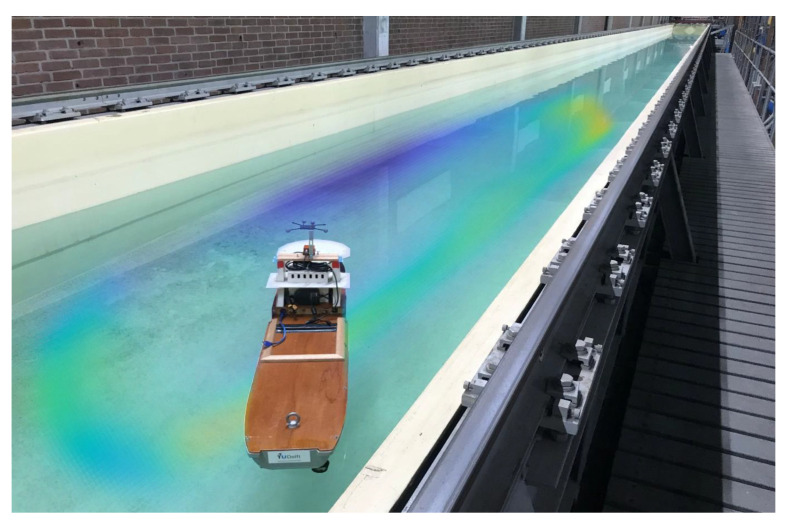
Learned magnetic field variations in test pool. The color corresponds to the estimated norm of the magnetic field map, while the opacity is inversely proportional with the variance of the estimate.

The contribution of this paper is an approach to magnetic field SLAM that is faster and requires less storage compared to the approach proposed in [8], inspired by the goal to run magnetic field SLAM in real-time on cheap carry-on units with low processing power. A property of using reduced-rank Gaussian process regression for magnetic field SLAM in a cubic domain is that the magnetic field map is given as a linear combination of analytically described basis functions [37]. We can therefore use the spatial derivatives of the closed-form solutions of the Laplacian to find the Jacobian of the magnetic field map with respect to the position estimate. To reduce computational expenses, we propose utilising the availability of analytical Jacobians of the reduced-rank Gaussian process magnetic field maps to perform magnetic field SLAM using an extended Kalman filter. This only requires building and updating a single copy of the magnetic field map at each time instance. Figure 1 shows the learned magnetic field map and estimated trajectory from our EKF algorithm for magnetic field SLAM, tested on magnetic field measurements collected onboard a model ship. The resulting computational cost is $O(N_{m}^{2})$ at each time step, instead of $O(N_{m}^{2}N_{p})$. The use of the EKF is possible if the dynamic model and measurement model are close to linear [38]. In the case of simultaneous localization and mapping, the world frame coordinate system is defined relative to the initial body frame coordinate system [8]. As there is no uncertainty in the initial position estimate due to this definition [8], the position estimate initially has zero covariance. In cases where the growth of the uncertainty of the pose estimate that comes from odometry drift is limited by frequent enough visitations of previous areas, magnetic field SLAM remedies drift in the position estimate, which means that the position estimation error no longer grows without bounds, but stays limited [8]. When the estimated position is close to the actual position, the magnetic field linearized about the estimated position provides a good local approximation to the magnetic field itself, as the magnetic field even in environments with strong stationary disturbances can be assumed to have limited spatial variability [8,11,20,39]. A key assumption for several implementations of estimating the magnetic field with GP regression is that the magnetic field in locations close to each other have a higher correlation than the magnetic field in locations that are further away [8,11,20,39]. In these implementations, how rapidly the correlation diminishes with increasing distance is encoded in a hyperparameter in the GP prior describing the length scale of the spatial variations in the magnetic field [39].

We illustrate with simulations that we can expect the EKF for a localization task to give accurate estimates when the max norm of the covariance from the predictive distribution is small relative to the length scale of the magnetic field anomalies. A requirement for using GP regression to represent the magnetic field map in magnetic field SLAM is the prior knowledge of hyperparameters describing the expected distribution of the magnetic field potential [8]. These hyperparameters contain information about the expected length scale of the magnetic field spatial variations [39]. Without adding any further assumptions to the magnetic field SLAM formulation presented in [8], we can therefore assume to have information available about how rapidly we can expect our learned magnetic field to vary spatially. In SLAM, the uncertainty of the position estimate will grow when the sensor moves through unexplored areas, as there is no map information available to correct the estimated pose [8]. When the sensor re-enters an area where it has already built a map of the local anomalies, our proposed algorithm can be expected to converge when the covariance of the position estimate is small compared to the length scale of the learned magnetic field map. We show with magnetic field data collected from a model ship in an indoor pool (see Figure 1) and simulated odometry that our proposed algorithm converges when the odometry noise is limited, for a trajectory when the time until the first revisitation of a mapped area is constant. We also show with magnetic field measurements and odometry obtained from an open-source implementation by [40] that our algorithm can compensate for drift in position estimates based on accelerometer and gyroscope measurements in a foot-mounted sensor.

The remainder of this paper is structured as follows. In Section 2, we define the model for our magnetic field SLAM estimation problem. In Section 3, we derive an EKF for magnetic field SLAM. In Section 4 we show the convergence properties of our algorithms in a simulated navigation task, where we can control the ratio of our position estimate uncertainty over the length scale of the magnetic field variations. In Section 5, we demonstrate the drift-compensating abilities of the EKF-SLAM algorithm on a set of data we collected with a model ship and on an open-source data-set from a foot-mounted sensor. Finally, in Section 6 we summarise our findings and discuss possible directions for future work. Our Matlab-implementation producing all results found in this paper can be found on https://github.com/fridaviset/EKFMagSLAM.

## 2. Modeling

Our simultaneous localization and mapping algorithm estimates the filtering distribution

(1) $$p(x_{t}|y_{1:t}^{b},\Delta p_{1:t}^{w},\Delta q_{1:t}^{b}),$$

where we denote the available magnetic field measurements by $y_{1:t}^{b}$, the odometry describing the change in position and orientation $\Delta p_{1:t}^{w},\Delta q_{1:t}^{b}$, and the state we wish to estimate at each time step *t* as $x_{t}$. For a vector $x_{t}$, the set $\left\{x_{1},\cdots ,x_{t}\right\}$ is denoted $x_{1:t}$ for brevity. We use the superscript b to denote the sensor’s body-frame, which is aligned with its sensor axes. The superscript w refers to the world frame, which is defined as the inertial frame that shares its origin with the body frame at time zero. The gravity field in this position is aligned with the negative *z*-axis, and where the initial yaw-angle between the body and world-frame at t=0 is zero. As we wish to estimate both position, orientation and the magnetic field map, we model our state as

(2) $$x_{t}={\left[{\left(p_{t}^{w}\right)}^{⊤}\;{\left(q_{t}^{wb}\right)}^{⊤}\;m^{⊤}\right]}^{⊤},$$

where $p_{t}^{w}$ denotes the position, $q_{t}^{wb}$ denotes the orientation transformation from the world frame to the body frame, and *m* is a vector that describes our magnetic field map represented with reduced-rank GP regression.

### 2.1. Measurement Model

We consider the case where we have access to measurements of the magnetic field in a sensor attached to the object we aim to localize. The measurement equation is given by

(3) $$y_{t}^{b}=R_{t}^{bw}\nabla_{p}\varphi \left(p_{t}^{w}\right)+e_{m,t}^{b},\;e_{m,t}^{b}∼N\left(0,\sigma_{m}^{2}I_{3}\right),$$

where $y_{t}^{b}$ denotes the magnetic field measurement, $e_{m,t}^{b}$ denotes the measurement noise and $R_{t}^{bw}$ denotes the rotation from world to body frame, corresponding to the conjugate of the quaternion $q_{t}^{wb}$, expressed as a rotation matrix. See [41] for definitions of the quaternion conjugate, and definitions of transformation from a quaternion to a rotation matrix. The function $\nabla_{p}\varphi \left(p_{t}^{w}\right)$ is our model of the magnetic field. We model the magnetic field as in [42] as the gradient of the function $\varphi (p_{t}^{w})$ with respect to the position $p_{t}^{w}$, where $\varphi :R^{3}\to R$ is a scalar potential distributed as a GP with prior

(4) $$\varphi ∼N(0,\kappa_{\mathrm{SE}}\left(\cdot ,\cdot \right)+\kappa_{\mathrm{lin}}\left(\cdot ,\cdot \right)),$$

and where the kernel is defined by the functions

(5a) $$\begin{array}{cc} \kappa_{\mathrm{SE}}\left(p,p^{\prime }\right) & =\sigma_{\mathrm{SE}}^{2}\exp\left(-\frac{∥p-p^{\prime }{∥}_{2}^{2}}{2l_{\mathrm{SE}}^{2}}\right), \end{array}$$

(5b) $$\begin{array}{cc} \kappa_{\mathrm{lin}}\left(p,p^{\prime }\right) & =\sigma_{\mathrm{lin}}^{2}p^{⊤}p^{\prime }, \end{array}$$

with $\sigma_{\mathrm{SE}}$, $\sigma_{\mathrm{lin}}$, $l_{\mathrm{SE}}$ and $\sigma_{m}$ being hyperparameters. The hyperparameter $l_{\mathrm{SE}}$ refers to the length scale of the spatial variations in the magnetic field potential that is represented by the kernel [42]. The parameters $\sigma_{\mathrm{SE}}$, $\sigma_{\mathrm{lin}}$ and $\sigma_{m}$ define the presence of the nonlinear components, linear components and measurement noise in the magnetic field respectively [42]. The linear component modelled by the kernel component $\kappa_{\mathrm{lin}}$ represents of the constant underlying earth magnetic field, while the nonlinear disturbances caused by the modelled by the nearby ferromagnetic structures is modelled by the kernel component $\kappa_{\mathrm{SE}}$. Modelling the magnetic field as the gradient of a scalar potential ensures that Maxwell’s equations are satisfied, under the assumption that no current passes through the domain where we construct our magnetic field map [42]. We use a reduced-rank approximation to the GP similar to the one used in [8] for mapping the indoor magnetic field for localization purposes with the same kernel. Our approach is an application of the GP approximation presented in [37], which is based on conditioning the GP prediction on a set of basis functions corresponding to a subset of the eigenbasis of the negative Laplace operator in a finite domain, subject to Dirichlet boundary conditions [37]. The reduced-rank approximation models the magnetic field potential as a sum of basis functions defined as the solutions to the Laplace equations over a finite domain $\Omega \subset R^{3}$

(6) $$\left\{\begin{array}{cc} -\nabla_{p}^{2}\phi_{i}\left(p\right)=\lambda_{i}\phi_{i}\left(p\right), & p\in \Omega ,\\ \phi_{i}\left(p\right)=0, & p\in \delta \Omega , \end{array}\right.$$

where $\phi_{i}$ is the *i*’th eigenfunction, and $\lambda_{i}$ is the *i*’th eigenvalue [37]. We approximate the GP with the first $N_{m}$ basis functions solving the Laplace equations defined over a cubical domain $\Omega =\left[L_{l,1},L_{u,1}\right]\times \left[L_{l,2},L_{u,2}\right]\times \left[L_{l,3},L_{u,3}\right]$. In this case, using the $N_{m}$ first eigenfunctions to represent the potential gives the approximation

(7) φ(p)≈Φ(p)m,

with Φ(p) being the matrix

(8) $$\Phi \left(p\right)=\left[\begin{array}{cccc} p^{⊤} & \phi_{1}\left(p\right) & \cdots & \phi_{N_{m}}\left(p\right) \end{array}\right],$$

where $\phi_{i}$ is the *i*’th eigenfunction of the Laplace basis, and $m\in R^{N_{m}+3}$ is a vector determining the contribution of each linear components as well as each basis function to the potential. Each eigenfunction $\phi_{i}\left(p\right)$ has a closed form expression given by

(9) $$\begin{array}{c} \phi_{i}\left(p\right)=\prod_{d=1}^{3}\frac{\sqrt{2}}{\sqrt{L_{u,d}-L_{l,d}}}\sin\left(\frac{\pi n_{i,d}\left(p_{d}+L_{l,d}\right)}{L_{u,d}-L_{l,d}}\right), \end{array}$$

where the set $\left(n_{i,1},n_{i,2},n_{i,3}\right)$ is the set of three natural numbers that is different from the sets $\left(n_{j,1},n_{j,2},n_{j,3}\right)$ defined for all j<i, that gives the corresponding eigenvalue

(10) $$\begin{array}{c} \lambda_{i}=\sum_{d=1}^{D}{\left(\frac{\pi n_{i,d}}{L_{u,d}-L_{l,d}}\right)}^{2}, \end{array}$$

as large as possible. The basis functions in (9) and eigenvalues in (10) are identical to those used in [22]. The vector *m* has a prior distribution given by

(11) m∼N(0,Λ),

where Λ is defined as

(12) $$\begin{array}{cc} \; & \Lambda =\mathrm{diag}\left[\sigma_{\mathrm{lin}}^{2}I_{3},\;S_{\mathrm{SE}}\left(\sqrt{\lambda_{1}}\right),\;\cdots ,\;S_{\mathrm{SE}}\left(\sqrt{\lambda_{N_{m}}}\right)\right], \end{array}$$

with $S_{\mathrm{SE}}\left(\cdot \right)$ being the spectral density of the squared exponential kernel, as defined in [7]. This corresponds to the magnetic field potential φ(p)≈Φ(p)m having a prior distribution given by (4) as $N_{m}$ goes to infinity, and the size of the domain goes to infinity [37]. Inserting this approximation to the magnetic field model gives the measurement model

(13) $$y_{t}^{b}\approx R_{t}^{bw}\nabla_{p}\Phi \left(p_{t}^{w}\right)m+e_{m,t}^{b}\;e_{m,t}^{b}∼N\left(0,\sigma_{m}^{2}I_{3}\right),$$

with the closed form expressions for $\nabla_{p}\Phi \left(p_{t}^{w}\right)$ given in Appendix A. This measurement model is identical to the measurement model used in [8], with the exception of the basis functions $\Phi (p_{t}^{w})$, which are different as they are defined with respect to different domains.

### 2.2. Dynamic Model

We assume access to noisy odometry measurements $\Delta p_{t}^{w}$ and $\Delta q_{t}^{b}$ of the change in position and orientation at each time step. We model the change in position and orientation according to the dynamic model

(14a) $$\begin{array}{cc} p_{t+1}^{w}=p_{t}^{w}+\Delta p_{t}^{w}+e_{p,t}^{w}, & e_{p,t}^{w}∼N\left(0,\sigma_{p}^{2}I_{3}\right), \end{array}$$

(14b) $$\begin{array}{ccc} q_{t+1}^{wb} & =q_{t}^{wb}⊙\Delta q_{t}^{b}⊙\exp_{q}\left(e_{q,t}^{b}\right), & e_{q,t}^{b}∼N\left(0,\sigma_{q}^{2}I_{3}\right), \end{array}$$

where $e_{p,t}^{w}$ and $e_{q,t}^{b}$ denote the position and orientation odometry measurement noise respectively, ⊙ is the quaternion product and $\exp_{q}$ is the operator that maps an axis-angle orientation deviation to a quaternion (see [41] for details on quaternion algebra).

## 3. Ekf for Magnetic Field Slam

We estimate our state with an EKF applied to the dynamic model in (14a,b) and the measurement model defined in (13), with predictive and filtered estimates denoted ${\hat{p}}_{t|t-1}^{w}$, ${\hat{m}}_{t|t-1}$, ${\hat{q}}_{t|t-1}^{wb}$ and ${\hat{p}}_{t|t}^{w}$, ${\hat{m}}_{t|t}$, ${\hat{q}}_{t|t}^{wb}$ respectively. We initialise the magnetic field state estimate as ${\hat{m}}_{0|0}=0_{N_{m}\times 1}$ according to the reduced-rank GP prior in (11). We initialise the orientation estimate according to the initial rotation ${\hat{q}}_{0|0}^{wb}=q_{0}^{wb}$ between the world and body frame as defined in Section 2. We initialise the position estimate as ${\hat{p}}_{0|0}^{w}=0_{3\times 1}$, also according to our definition of the world frame relative to the initial body frame from Section 2.

We represent the deviation between the true and estimated predictive state by an error state $\xi_{t}$ defined as

(15) $$\begin{array}{cc} \xi_{t} & ={\left[{\left(\delta_{t}^{w}\right)}^{⊤}\;{\left(\eta_{t}^{w}\right)}^{⊤}\;\nu_{t}^{⊤}\right]}^{⊤}, \end{array}$$

where $\delta_{t}^{w}=p_{t}^{w}-{\hat{p}}_{t|t-1}^{w}$ denotes the position estimation error, $\nu_{t}=m-{\hat{m}}_{t|t-1}$ denotes the magnetic field state estimation error, and where $\eta_{t}^{w}$ denotes the orientation estimation error parametrised as an axis-angle deviation according to

(16) $$\begin{array}{cc} q_{t}^{wb}= & \exp_{q}\left(\eta_{t}^{w}\right)⊙{\hat{q}}_{t|t-1}^{wb}. \end{array}$$

Similarly, we represent the deviation between the true and estimated filtered state by an error state ${\tilde{\xi }}_{t}$ defined as

(17) $$\begin{array}{cc} {\tilde{\xi }}_{t} & ={\left[{\left({\tilde{\delta }}_{t}^{w}\right)}^{⊤}\;{\left({\tilde{\eta }}_{t}^{w}\right)}^{⊤}\;{\tilde{\nu }}_{t}^{⊤}\right]}^{⊤}, \end{array}$$

where ${\tilde{\delta }}_{t}^{w}=p_{t}^{w}-{\hat{p}}_{t|t}^{w}$, ${\tilde{\nu }}_{t}=m-{\hat{m}}_{t|t}$, and where ${\tilde{\eta }}_{t}^{w}$ denotes the filtered orientation estimation error according to

(18) $$\begin{array}{cc} q_{t}^{wb}= & \exp_{q}\left({\tilde{\eta }}_{t}^{w}\right)⊙{\hat{q}}_{t|t}^{wb}. \end{array}$$

Since we build our map relative to our initial position and orientation, the covariance of our initial position and orientation estimates is zero. The covariance of the initial magnetic field estimate is defined in (11) as the magnetic field map prior Λ. Hence, our initial error state $\xi_{0}$ has a covariance

(19) $$\begin{array}{c} P_{0|0}=\left[\begin{array}{cc} 0_{6\times 6} & 0_{6\times (N_{m}+3)}\\ 0_{(N_{m}+3)\times 6} & \Lambda \end{array}\right]. \end{array}$$

To perform the dynamic update, we propagate our filtered state estimate through the nonlinear dynamic model (14a,b), giving the predictive updates as described in (26a,b). As the magnetic field is assumed stationary, its estimate is unchanged by the dynamic update defined in (26c). We derive the covariance update in the EKF by linearising about the filtered state estimate from the previous time step, with respect to the error state ${\tilde{\xi }}_{t}$. Inserting (15), (17) and (26a,b) into the dynamic model (14a,b) gives

(20a) $$\begin{array}{cc} {\hat{p}}_{t|t-1}^{w}+\delta_{t}^{w} & ={\hat{p}}_{t-1|t-1}^{w}+\Delta p_{t}^{w}+{\tilde{\delta }}_{t-1}^{w}+e_{p,t}^{w} \end{array}$$

(20b) $$\begin{array}{cc} \exp_{q}\left(\eta_{t}^{w}\right) & =\exp_{q}\left({\tilde{\eta }}_{t-1}^{w}\right)⊙{\hat{q}}_{t|t-1}^{wb}⊙\exp_{q}\left(e_{q,t}^{b}\right)⊙{\left({\hat{q}}_{t|t-1}^{wb}\right)}^{C}, \end{array}$$

where ${\left({\hat{q}}_{t|t-1}^{wb}\right)}^{C}$ denotes the conjugate of the quaternion ${\hat{q}}_{t|t-1}^{wb}$. The linearization of the dynamic model with respect to the error states gives the following propagation of the error states

(21a) $$\begin{array}{cc} \delta_{t}^{w} & ={\tilde{\delta }}_{t-1}^{w}+e_{p,t}^{w}, \end{array}$$

(21b) $$\begin{array}{cc} \eta_{t}^{w} & \approx {\tilde{\eta }}_{t-1}^{w}+{\hat{R}}_{t|t-1}^{wb}e_{q,t}^{b}, \end{array}$$

(21c) $$\begin{array}{cc} \nu_{t} & ={\tilde{\nu }}_{t-1}, \end{array}$$

where ${\hat{R}}_{t|t-1}^{wb}$ denotes the rotation matrix corresponding to the rotation represented by the quaternion ${\hat{q}}_{t|t-1}^{wb}$. This linearization is exact for the position, and it is a good approximation for the orientation error state in the cases where the orientation error is small [41]. Equation (21a,b) can equivalently be written as

(22) $$\xi_{t}\approx {\tilde{\xi }}_{t-1}+e_{\mathrm{dyn},t},\;e_{\mathrm{dyn},t}∼N\left(0_{(N_{m}+9)\times 1},Q\right),$$

where

(23) $$Q=\left[\begin{array}{ccc} \sigma_{p}^{2}I_{3} & 0_{3\times 3} & 0_{3\times (N_{m}+3)}\\ 0_{3\times 3} & \sigma_{q}^{2}I_{3} & 0_{3\times (N_{m}+3)}\\ 0_{(N_{m}+3)\times 3} & 0_{(N_{m}+3)\times 3} & 0_{\left(N_{m}+3\right)\times \left(N_{m}+3\right)} \end{array}\right].$$

As the linearization of the error state propagation is given in (22), the covariance $P_{t|t-1}$ of the predictive state error $\xi_{t}$ is given by (26d).

For the measurement update, we apply an EKF measurement update to the measurement model in (13). We linearise about the predictive state estimate, with respect to the error state $\xi_{t}$. The linearized measurement model is given by

(24) $$y_{t}^{b}={\hat{R}}_{t|t-1}^{bw}\nabla_{p}\Phi \left({\hat{p}}_{t|t-1}^{w}\right){\hat{m}}_{t}+H_{t}\xi_{t}+e_{m,t}^{b},\;e_{m,t}^{b}∼N\left(0_{3\times 1},\sigma_{m}^{2}I_{3}\right),$$

where

(25) $$H_{t}={\left[\begin{array}{c} {\left(\nabla_{\mathrm{pp}}\Phi \left({\hat{p}}_{t|t-1}^{w}\right){\hat{m}}_{t}\right)}^{⊤}\\ {\left[\left(\nabla_{p}\Phi \left({\hat{p}}_{t|t-1}^{w}\right){\hat{m}}_{t}\right)\times \right]}^{⊤}\\ \left(\nabla_{p}\Phi {\left({\hat{p}}_{t|t-1}^{w}\right)}^{⊤}\right) \end{array}\right]}^{⊤}$$

with $\left[v\times \right]$ being the scew-symmetric matrix representing the cross product v×u between two vectors $v,u\in R^{3}$ as a matrix multiplication $\left[v\times \right]u$ (explicit expression for $\left[v\times \right]$ is given in [41]), and $\nabla_{\mathrm{pp}}\Phi \left(\cdot \right)$ being the Jacobian of the basis functions, given in Appendix A. Note that this Jacobian is a matrix with $3\times 3\times (N_{m}+3)$ entries, and multiplying it with the $\left(N_{m}+3\right)$-dimensional state vector *m* therefore gives a 3×3 matrix. Applying the Kalman filter measurement update to this linearized measurement function gives the EKF measurement update in (27a–e). This gives an estimate ${\hat{\xi }}_{t}$ of the predictive state error $\xi_{t}$, with a corresponding covariance. By concatenating the estimated predictive state error to the predictive state, we obtain the filtered state estimates as defined in (28a–c). The covariance of the filtered error state ${\tilde{\xi }}_{t}$ then becomes the same as the covariance of the estimated predictive error state ${\hat{\xi }}_{t}$. Recursively applying the dynamic update, measurement update and re-linearization step results in Algorithm 1.


**Algorithm 1:** EKF for magnetic field SLAM
   Input: ${\left\{\Delta p_{t}^{w},\Delta q_{t}^{b},y_{t}^{b}\right\}}_{t=1}^{N}$
   Output: ${\left\{{\hat{p}}_{t|t}^{w}\right\}}_{t=1}^{N}$, ${\left\{{\hat{q}}_{t|t}^{wb}\right\}}_{t=1}^{N}$, ${\left\{{\hat{m}}_{t|t}\right\}}_{t=1}^{N}$
          *Initialisation*: ${\hat{p}}_{0|0}^{w}=0_{3\times 1}$, ${\hat{q}}_{0|0}^{wb}=q_{0}^{wb}$, ${\hat{m}}_{0|0}=0_{(N_{m}+3)\times 0}$, (19)
1: **for** t=1 to *N* **do**
2:    Dynamic update
(26a)$${\hat{p}}_{t|t-1}^{w}={\hat{p}}_{t-1|t-1}^{w}+\Delta p_{t}^{w}$$
(26b)$${\hat{q}}_{t|t-1}^{wb}={\hat{q}}_{t-1|t-1}^{wb}⊙\Delta q_{t}^{b}$$
(26c)$${\hat{m}}_{t|t-1}={\hat{m}}_{t-1|t-1}$$
(26d)$$P_{t|t-1}=P_{t-1|t-1}+Q$$

3:    Measurement update
(27a)$$z_{t}={\hat{R}}_{t|t-1}^{wb}y_{t}^{b}-\nabla \Phi_{p}\left({\hat{p}}_{t|t-1}^{w}\right){\hat{m}}_{t|t-1}$$
(27b)$$S_{t}=H_{t}P_{t|t-1}H_{t}^{⊤}+\sigma_{m}^{2}I_{3}$$
(27c)$$K_{t}=P_{t|t-1}H_{t}^{⊤}S_{t}^{-1}$$
(27d)$${\hat{\xi }}_{t}=K_{t}z_{t}$$
(27e)$$P_{t|t}=P_{t|t-1}-K_{t}S_{t}K_{t}^{⊤}$$
4:    Relinearization
(28a)$${\hat{p}}_{t|t}^{w}={\hat{p}}_{t|t-1}^{w}+{\hat{\delta }}_{t}^{w}$$
(28b)$${\hat{q}}_{t|t}^{wb}=\exp_{q}\left({\hat{\eta }}_{t}^{w}\right)⊙{\hat{q}}_{t|t-1}^{wb}$$
(28c)$${\hat{m}}_{t|t}={\hat{m}}_{t|t-1}+{\hat{\nu }}_{t}$$
5: **end for**



## 4. Simulations

We study when Algorithm 1 for localization in a previously learned magnetic field gives a converging pose estimate in a known nonlinear field depending on the position uncertainty at time *t*. Since we assume the magnetic field map is know, we can replace ${\hat{m}}_{t|t}$ with the known $m_{\mathrm{sim}}$ everywhere in our algorithm. As a consequence of this, we can alo skip (26c) and (28c), use $P_{0|0}=0_{6\times 6}$ and

(29) $$H_{t}={\left[\begin{array}{c} {\left(\nabla_{\mathrm{pp}}\Phi \left({\hat{p}}_{t|t-1}^{w}\right)m\right)}^{⊤}\\ {\left[\left(\nabla_{p}\Phi \left({\hat{p}}_{t|t-1}^{w}\right)m\right)\times \right]}^{⊤} \end{array}\right]}^{⊤}.$$

We start the simulation at time *t* with a varying predictive position estimation error, and set the standard deviation of our position uncertainty equal to the distance between the actual and estimated position at the beginning of the simulation. This artificially introduces a predictive position estimation error representing the estimation error that can accumulate over time in magnetic field SLAM. Position errors can, for example, accumulate when the sensor is moved for a long time through areas with no information about the magnetic field available from previous measurements [8].

We simulate positions $p_{t}$ along a square trajectory moving with constant velocity for four laps, and simulate the odometry by adding sampled realisations of the white noises $e_{p,t}^{w}$ and $e_{q,t}^{b}$ to (14a,b). We simulate a nonlinear field by drawing a sample from the reduced-rank GP prior $m_{\mathrm{sim}}∼N\left(0,\Lambda \right)$, with $\sigma_{\mathrm{lin}}=0$, $\sigma_{\mathrm{SE}}=0.1$ and $l_{\mathrm{SE}}=0.2$. We used 50 basis functions to represent the magnetic field map. Using a domain that is 3m×3m×1m. This number of basis functions ensures that for a GP trained with 2000 sampled measurements the root mean squared error (RMSE) between the approximate and the full GP predictions in 1000 randomly selected locations is below the measurement noise. To prevent ill effects from the boundary conditions we ensured that both the training and test data was at least 0.5 m away from the border. We then simulate magnetic field measurements by adding white noise to the gradient of the nonlinear field in the ground truth position according to (13), replacing ${\hat{m}}_{t}$ with $m_{\mathrm{sim}}$, and using $\sigma_{m}=0.03$. We use the incoming magnetic field measurements and the odometry to estimate the position and orientation using an EKF and a particle filter. The EKF is implemented according to Algorithm 1, but reducing the state-space to only contain the position and orientation, and inserting $m_{\mathrm{sim}}$ in place of ${\hat{m}}_{t}$ in (27a). We implement a particle filter for navigation in a magnetic field represented by a field learned with GP regression according to Algorithm 1 in [20], with the difference that we use the simulated reduced-rank map instead of a learnt full GP map, and that we perform the prediction step using our odometry model in (14a,b).

**Figure 2:**
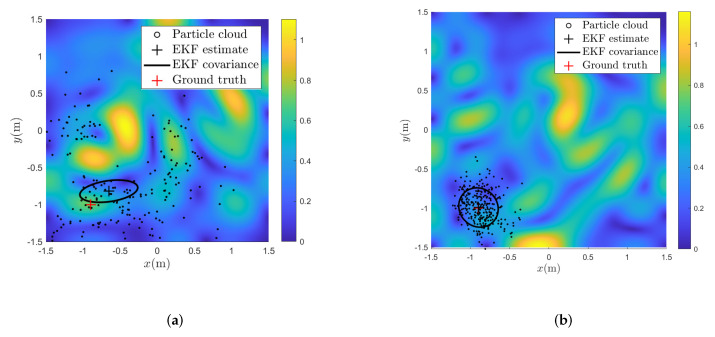
Comparison of approximations of the filtered position distribution given measurements from a simulated nonlinear field. The color indicates the norm of the simulated magnetic field. The covariance ellipsoids indicate the 68% confidence interval of the EKF estimate. (**a**) Estimates of the filtered distribution based on predictive estimates with error 0.40 m. (**b**) Estimates of the filtered distribution based on predictive estimates with error 0.05 m.

**Figure 3:**
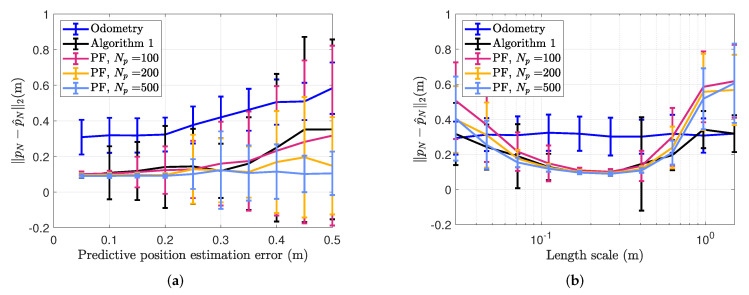
Simulation, investigating drift-compensating abilities given varying predictive position estimation errors. Comparison of position estimation error at the end of the trajectory between Algorithm 1 and a particle filter for localization in a known map with varying predictive position errors at the initialisation of the simulation. The lines connect the average results after 100 Monte Carlo repetitions with different realisations of the odometry noise, and the error bars represent one standard deviation. (**a**) Estimation accuracies with varying predictive position error. (**b**) Estimation accuracies with varying length scales $l_{\mathrm{SE}}$.

In Figure 2, we can see two examples of PFs and an EKFs estimate of the position filtered distribution, represented with a particle cloud and a mean and an uncertainty interval, respectively. In Figure 2a, the initial uncertainty of the position estimate is so large that the particle cloud becomes multi-modal, making it impossible for the EKF to correctly approximate the true nature of the filtered distribution. The estimated position is, therefore, far away from the true position. In addition, the uncertainty estimate of the EKF does not reflect this, as it relies upon a linearization of the nonlinear magnetic field about the predictive estimate. In Figure 2b, the position estimate at time *t* is still wrong, but close enough that the particle cloud representation of the filtered distribution appears uni-modal, and the EKF estimate of the filtered distribution is now closer to the estimate from the particle filter. As we see in Figure 3a displaying the position estimation error at the end of the trajectory estimates across the four laps, the position estimates from the EKF are accurate across the entire trajectory if the predictive position error is lower than 0.3 m. The particle filter on the other hand, is accurate even beyond these predictive position accuracies when using 500 particles, while it is only slightly improving the prediction accuracy over Algorithm 1 when using 100 or 200 particles. The accuracy is better for 200 particles than for 100 particles. For only 100 particles, the average prediction accuracy is worse than for Algorithm 1 for a predictive position accuracy of 0.2 m, likely due to the fact that the particle filter is a Monte-Carlo method, meaning that there is never a guarantee for convergence [43]. In Figure 3b, the average estimation accuracy for varying length scales of the simulated magnetic field is displayed. As in Figure 3a, the performance of the particle filter improves with increasing amount of particles. Algorithm 1 is able to compensate for odometry drift and achieve estimation error on average below 0.2 m for length scales between 0.1 and 0.4 m, using a constant predictive position error of 0.1 m. For length scales below 0.1 m, the linearization error becomes too big for the approximation accuracy of our linearized model to give a good result. For length scales higher than 0.4 m, the variations in the magnetic field are not rich enough to provide valuable information about the position of the sensor. Therefore, as the length scale of the field increases, even though the field becomes closer to linear and the linearization error continues to decrease, the estimation accuracy does not improve—because the signal-to-noise ratio from the magnetic field measurements also decreases. As we for the simulation results in Figure 3a use a simulated magnetic field map with spatial variations of 0.3 m, this indicates that as long as the covariance of the predictive distribution does not exceed the length scale of the magnetic field, we can expect Algorithm 1 to have the same estimation accuracy as particle-based methods.

## 5. Experimental Results

### 5.1. Model Ship Experiments

**Figure 4:**
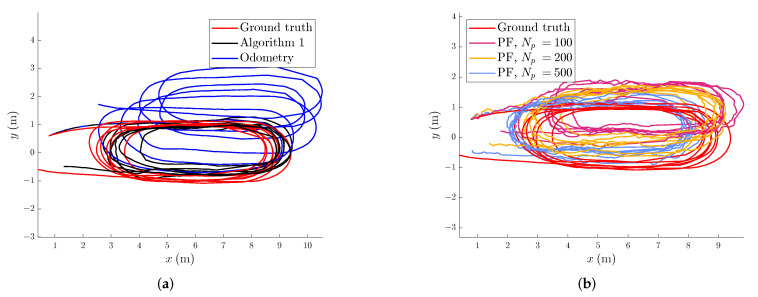
Comparison of the model ship position trajectory estimates for a single realisation of simulated odometry noise from a birds-eye view. (**a**) Comparing Algorithm 1 and the odometry to the ground truth. (**b**) Comparison of the position estimates from the RBPF with 100, 200 and 500 particles respectively to the ground truth.

We performed experiments to test Algorithm 1 on a model ship in a pool. The magnetic field on the model ship was measured using an Xsens MTi-100 Inertial Measurement Unit (IMU). We recorded the ground truth position and orientation using a motion capture system with cameras and optical markers mounted on the model ship, as shown in Figure 1. The motion capture markers and the IMU were rigidly attached to the ship. The IMU measurements were collected on a computer onboard the ship. The magnetic field was disturbed by metal railings and building structures near the pool. We steered the model ship around in long loops in the pool, with a ground truth trajectory that is displayed in red in Figure 4. The magnetic field measurements were collected in the IMU at 200 Hz, and down-sampled to 5 Hz. The odometry was simulated based on the ground truth position and orientation, according to the odometry model in (14a,b), also at a frequency of 5 Hz (but with some motion capture measurement dropouts due to pool reflections). We simulate drifting odometry by computing the change in position and orientation at each time step from the ground truth and adding a simulated white noise with standard deviation $\sigma_{p}=0.01$, $\sigma_{q}=0.001$. For these experiments, we use real magnetic field data and simulated odometry to investigate the effects of changing odometry noise on our algorithm. In addition, we simulate a constant position odometry bias of [0.0030.0030] m/time step. Our algorithm was not originally designed to compensate for constant position odometry biases. However, as this often occurs in practice (for example, when the odometry sensors are not perfectly calibrated), we chose to include it in our simulated odometry to test our algorithms’ ability to compensate for a drift that consists both of integrated white noise and a constant disturbance.

**Figure 5:**
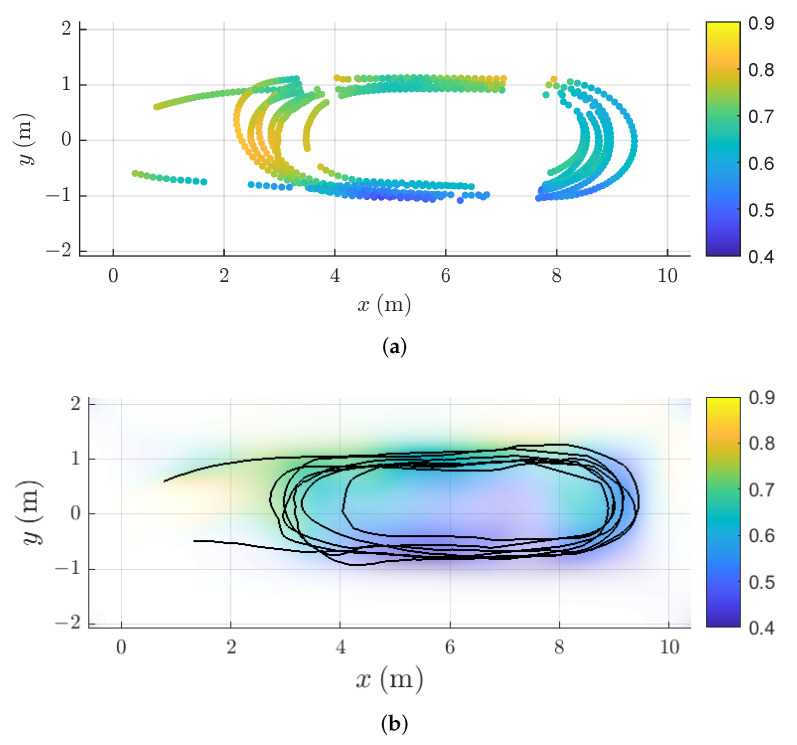
Measured and estimated magnetic field and position trajectories for the model ship. The upper plot marks with circles the locations where magnetic field measurements were successfully collected and matched with a ground truth position in the model ship, and the colors of the circles correspond to the norm of the measured magnetic field. The lower plot displays the trajectory estimate from applying Algorithm 1 in black. It also shows the learned magnetic field map, where the color corresponds to the norm of the estimated magnetic field $∥\nabla_{p}\Phi \left(p\right){\hat{m}}_{N|N}{∥}_{2}$, and the opacity is inversely proportional with the trace of the covariance matrix of the magnetic field map estimate in each location, $\mathrm{Tr}(\nabla_{p}\Phi \left(p\right)P_{N|N}{\left(\nabla_{p}\Phi \left(p\right)\right)}^{⊤})$. (**a**) Measured magnetic field norm in ground truth positions. (**b**) The estimated magnetic field norm is displayed with the semi-transparent color map and the estimated trajectory is displayed with the black line.

In magnetic field SLAM, there is usually no possibility of optimising the hyperparameters for GPs prior to estimating the map, as there is no magnetic field data available. This motivates us to choose hyperparameters prior to running Algorithm 1 [8]. The hyperparameters were set to $l_{\mathrm{SE}}=0.8$, $\sigma_{\mathrm{SE}}^{2}=1$, $\sigma_{m}^{2}=0.01$, $\sigma_{\mathrm{lin}}^{2}=1$ and 50 basis functions. The basis functions were defined with respect to a cubical domain Ω which is as small as possible, and whose border is at least 1 m away from the closest ground truth position. We confirmed empirically that 50 basis functions is sufficient to ensure that the RMSE between the approximation and the full GP predictions in 1000 randomly selected locations in the domain is below the measurement noise. We based the predictions on 2000 samples from the full GP prior, sampled from randomly selected locations in the domain at least 1 m away from the domain border. For the first set of experiments, we investigate and compare the position estimation error of Algorithm 1 with the particle filter-based approach to magnetic field SLAM [8] for odometry noise levels of $\sigma_{p}=0.01$, $\sigma_{q}=0.001$. In Figure 5a, the norm of the magnetic field measurements in locations where there were no motion-capture dropouts are displayed. The norm of the measured magnetic field ranges between 0.46 and 0.83 (the Xsens MTi-100 provides unit-less measurements proportionatal to the magnetic field strength). The spatial variations in the measured magnetic field norm are visible in Figure 5a, and the magnetic field stays close to constant for position changes of less than 0.1 m, while it can change as much as from 0.46 to 0.83 when the position change is more than 1 m. As the spatial variations in the magnetic field potential are the sources of the spatial variations in the magnetic field norm [39], we expect that our assumed length scale of 0.8 m is close enough to the actual length scale of the magnetic field variations to allow for Algorithm 1 to compensate for position estimation drift in the odometry. Figure 5b shows the norm of the magnetic field map learned by Algorithm 1, and comparing to the measured magnetic field norms in Figure 5a, we can see that the norm of the magnetic field estimates and the estimated trajectory are similar, with the learned magnetic field map prediction being more certain in and near the areas where there are more magnetic field measurements available. In Figure 4a, the estimated trajectory from Algorithm 1 is compared with the ground truth trajectory, as well as the dead reckoning position estimate from the simulated odometry. The position estimate from Algorithm 1 compensates visibly for the drift in the odometry.

**Figure 6:**
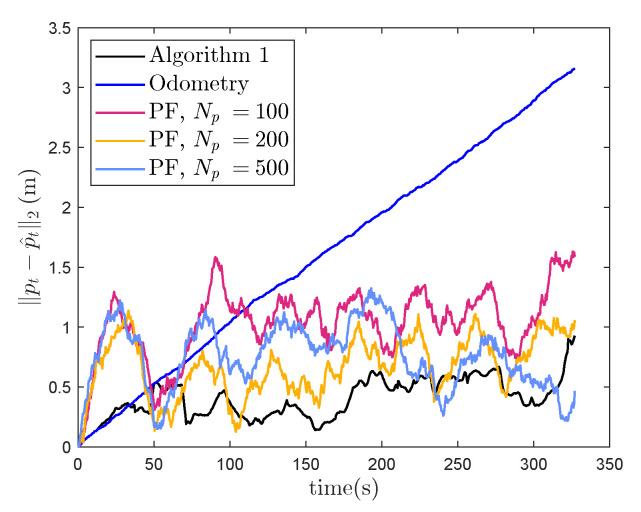
Comparison of model ship position estimation errors from Algorithm 1, drifting odometry and the PF with 100, 200 and 500 particles respectively for a single realisation of simulated odometry noise.

In Figure 6 the position estimation error of the odometry and Algorithm 1 can be seen to increase at the beginning of the trajectory. After around 31 s, Algorithm 1 can use the learned magnetic field map in combination with the incoming magnetic field measurements to compensate for drift in the estimated position and orientation. In Table 1, the position estimation RMSE values for 4 collected data sets of a similar shape as the one displayed in Figure 4a is shown after repeated experiments with the same odometry noise and the same constant drift in the xy-plane, showing that the reduction of RMSE is comparable also for repeated experiments. The position estimation error of the dead reckoning can increase potentially unbounded, while the position estimation error of Algorithm 1 remains bounded when the ship revisits previously mapped areas. However, it will only remain bounded if the quality of the map is good enough to provide information to the position estimate. In Table 2, the runtime for each of the algorithms is displayed. The runtime of the PF methods grows proportionally with the number of particles used. As can be seen in Table 1, the PF performs on average worse than the EKF. The trajectory of the highest-weight particle from a single run of each of the PFs is shown in Figure 4b. From our simulation results, given enough particles, the particle filter compensates for drift at least as well as Algorithm 1. However, in contrast to our simulation results where we investigate how well the particle filter performs localization given a previously learned map, in practice, the particle filter has to rely upon magnetic field maps created conditionally on each particle. From these results a standard implementation of the PF in [8] with cubic domain basis functions and resampling at every timestep, with the same hyperparameter settings as the EKF performs worse on our collected model ship data, even given 500 particles. In general, the performance of the particle filter can depend on the resampling strategy, the measurement noise and the process noise [44]. Another possible explanation why the particle filter performs worse for the full SLAM scenario compared to the simulation case, is the fact that for long trajectories, the resampling step can cause loss of diversity amount the particles [45].

**Figure 7:**
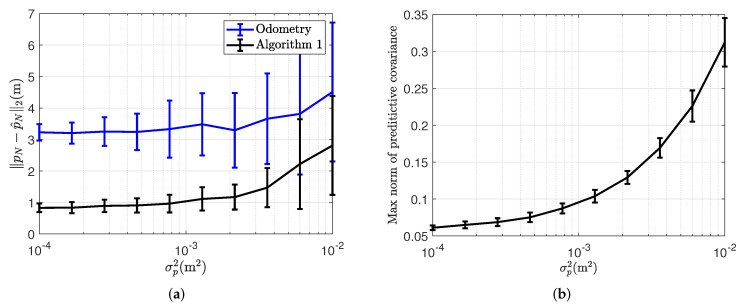
Investigation of the effect of varying odometry noise on the model ship position estimate. The lines connect the average results of the ship position estimation after 100 Monte Carlo repetitions with different realisations of the simulated odometry for varying amounts of odometry noise. (**a**) Model ship position estimation error at the end of the trajectory for varying amounts of odometry noise. (**b**) The max norm of the predictive covariance of the estimate from Algorithm 1 depending on varying odometry noise.

The linearization of the measurement function in (13) is performed around the predictive position estimate. The covariance of the estimate can grow when the sensor is moved through an area where the map is previously unknown. The growth rate will depend on the odometry noise. This is demonstrated in Figure 7b, where for 100 Monte Carlo simulations with different odometry noise realisations, the max norm of the predictive estimate from Algorithm 1 can be seen to increase with increasing odometry noise for the same trajectory. In Figure 7a, it can be seen that if we increase the simulated odometry noise above $\sigma_{p}^{2}=0.002$, the position estimation error of Algorithm 1 is no longer able to compensate for drift in the dead reckoning. A higher odometry noise means more drift is likely to accumulate to the predictive estimation error before revisiting a previously mapped area. The inability of Algorithm 1 to compensate for drift caused by odometry noises above $\sigma_{p}^{2}=0.002$, therefore, reflects how the assumptions of the measurement function being locally linear no longer hold when the covariance of the predictive distribution becomes large compared to the length scale of the magnetic field disturbances. For the experimental results, for odometry noises above $\sigma_{p}^{2}=0.002$, we observe a predictive covariance max norm of 0.15 m in the results in Figure 7b and an accumulated drift of 0.5 m, which is combination is comparable in magnitude to our length scale $l_{\mathrm{SE}}$ of 0.8 m. These results are comparable to our simulation results in Section 4, where Algorithm 1 for localization only converges when the position error is 0.3 m using a length scale of 0.2 m. In both cases, when the order of the prediction error goes beyond the length scale of the magnetic field variations, Algorithm 1 is no longer able to compensate for drift in the position estimate.

### 5.2. Magnetic Field Slam for Pedestrians with Foot-Mounted Sensor

Using accelerometer and gyroscope measurements from an IMU mounted on the foot of a pedestrian, it is possible to estimate the position of the pedestrian with high accuracy on a short timescale using a zero-velocity-update (ZUPT) aided EKF [46]. The estimate is obtained by integrating the change in orientation and velocity. In addition, the assumption that when the foot is in the stationary part of the step, it has zero velocity is used to reduce the drift of the position and orientation estimates [46]. The position and orientation estimates are typically accurate at the beginning of a trajectory but can drift over time if biases and/or white noise affect the measurements [47]. This filter was implemented in an open-source implementation by [47].

**Figure 8:**
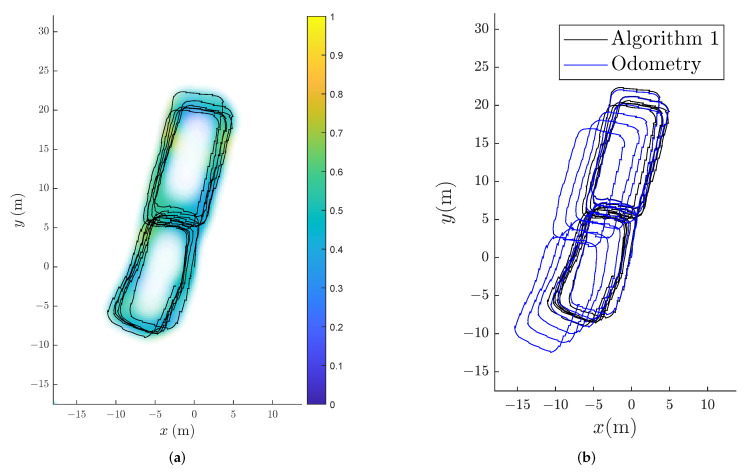
Trajectory and magnetic field map estimate for the foot-mounted sensor data. The estimated trajectory obtained with Algorithm 1 is compared to odometry from the foot-mounted sensor data obtained via [40] implementation of the ZUPT-aided EKF using a foot-mounted accelerometer and gyroscope. The color of the magnetic field map corresponds to the norm of the estimated magnetic field, and the opacity is inversely proportional with the sum of the marginal variance for each of the three estimated magnetic field components. (**a**) Learned magnetic field displayed with the semi-transparent color map and estimated trajectory displayed with the black line with odometry from foot-mounted sensor from a birds eye view. (**b**) Trajectory estimate from Algorithm 1 compared to odometry from a birds eye view.

To test the capabilities of Algorithm 1 on measurements from a foot-mounted sensor, we used magnetic field measurements present in the open-source data set used in [40], to remedy the drift present in the position estimates obtained form their ZUPT-aided EKF. The data set from [40] contains multiple measurement series from an IMU collected in the same hallway, walking the same trajectory. Although the implementation in [40] only used the accelerometer and gyroscope measurements from the IMU, magnetic field measurements were also collected, and are included in the published data. We first ran the open-source implementation from [40] of the ZUPT-aided EKF on the 12 available measurement sequences that were made by collected while walking in a similar trajectory. We ran the ZUPT-aided EKF independently on the 12 experiments and obtained 12 sets of position and orientation estimates. We then concatenated the 12 estimated trajectories by initialising each trajectory at the position and orientation where the previous trajectory ended. This gave a drifting odometry estimate of the position of the pedestrian. The drifting odometry is displayed in Figure 8b. This odometry has an increasing error in position and orientation over time partly because the ZUPT-aided EKF will have some position and orientation drift inherently and partly because of the assumption that the foot-mounted sensor ends in the same orientation at the end of each collected data set as the beginning of the next data set may not be exactly true. However, we can see that most drift accumulates in a constant direction, and drift caused by wrong orientation initialisation should cause twisting of the trajectory. It is, therefore, likely that most of the drift visible in Figure 8b is present due to inherent drift in the ZUPT-aided EKF. To use the odometry in Algorithm 1, we down-sampled the position and orientation estimates to 10 Hz and computed the change in position and orientation between each time step. We then used the changes in position and orientation as input odometry. As SLAM is performed in real-time, we cannot know the hyperparameters a priori to running the algorithm. The magnetic field measurements available in the open-source data set from [40] were without reported units but had a norm that ranged between 0.2429 and 0.8584. We therefore selected the expected nonlinear variations $\sigma_{\mathrm{SE}}^{2}=1$. We assumed that the contribution from the constant earth magnetic field had approximately the same order of magnitude and so selected $\sigma_{\mathrm{lin}}^{2}=1$. We set the length scale to $l_{\mathrm{SE}}=2$ m, and we set the measurement noise to be $\sigma_{m}^{2}=0.01$. We used 1850 basis functions to approximate the magnetic field map. We selected a domain which was the smallest possible cube that was still at least 10 m away from the first lap of the odometry. We found empirically that 1850 were a sufficient amount of basis functions using the same approach as in Section 4 and Section 5. The resulting position estimate from Algorithm 1 compensates for drift in the odometry, as shown in Figure 8a.

## 6. Conclusions and Future Work

We proposed using an EKF for magnetic field SLAM, which is computationally more efficient and requires less memory than previously proposed methods for magnetic field SLAM. Promisingly, we demonstrated that our proposed algorithm compensates for odometry drift in a way that is comparable to previously proposed, more computationally expensive methods. Using an experiment with magnetic field measurements collected onboard a model ship and using simulated odometry, we ran Monte-Carlo simulations investigating the capabilities of our algorithm to compensate for odometry drift for varying amounts of odometry noise, illustrating that when the uncertainty of the estimate is small compared to the length scale of the magnetic field variations, our proposed algorithm will give a position estimate that compensates for drift in odometry. We also demonstrated the abilities of our proposed algorithm to compensate for drift on an open-source data-set collected with a foot-mounted sensor.

To employ our proposed algorithm in real-life applications such as indoor, surface, underground or underwater navigation, it would be necessary to incorporate sources of odometry information that are available in real-life scenarios, such as inertial sensors or visual odometry from cameras. Another possible direction of future work could be to implement an iterated EKF or another extended Kalman-filter based estimation method that can handle larger non-linearities compared to the EKF [48], and investigate if this improves the convergence of the method. Future research could also look into further reducing the computational requirements associated with reduced-rank GP regression.

## Figures and Tables

**Table 1:** Trajectory RMSE values in meters for the 4 collected data sets from the model ship. Values are given as averages ± one standard deviation, after 100 Monte Carlo repetitions with different realisations of the simulated odometry noise.

Trajectory RMSEs	Data Set 1	Data Set 2	Data Set 3	Data Set 4
Algorithm 1	0.53 ± 0.15	0.58 ± 0.18	0.53 ± 0.24	0.98 ± 0.62
RBPF with 100 particles	0.85 ± 0.27	0.92 ± 0.26	0.95 ± 0.42	1.53 ± 0.53
RBPF with 200 particles	0.85 ± 0.22	0.89 ± 0.27	0.98 ± 0.47	1.48 ± 0.46
RBPF with 500 particles	0.87 ± 0.19	0.86 ± 0.24	1.00 ± 0.40	1.53 ± 0.50
Odometry	1.98 ± 0.54	1.52 ± 0.48	1.76 ± 0.52	1.65 ± 0.47

**Table 2:** Measured time to run the estimation algorithm (in seconds) for the 4 collected data sets from the model ship. Values are given as averages ± one standard deviation, after 100 Monte Carlo repetitions with different realisations of the simulated odometry noise.

Runtimes	Data Set 1	Data Set 2	Data Set 3	Data Set 4
Algorithm 1	0.06 ± 0.01	0.05 ± 0.00	0.14 ± 0.01	0.05 ± 0.00
RBPF with 100 particles	12.85 ± 0.26	9.24 ± 0.10	21.55 ± 0.32	9.91 ± 0.14
RBPF with 200 particles	25.70 ± 0.49	18.44 ± 0.166	42.64 ± 0.27	19.72 ± 0.20
RBPF with 500 particles	64.24 ± 0.82	46.02 ± 0.20	106.93 ± 0.34	49.43 ± 1.29

## Data Availability

The data used to obtain the results in this paper can be found in https://github.com/fridaviset/EKFMagSLAM.

## Abbreviations

The following abbreviations are used in this manuscript:

SLAM	Simultaneous localization and mapping
EKF	Extended Kalman filtering
RBPF	Rao-Blackwellized particle filter
GP	Gaussian process
IMU	Inertial measurement unit
ZUPT	Zero-velocity upate
RMSE	Root mean squared error
GNSS	Global navigation satelite system

## Appendix A. Analytical Jacobians

The gradient of the basis functions $\nabla_{p}\Phi \left(p\right)$ used in (13) (the basis functions Φ(p) are defined in (8)) is a $3\times (N_{m}+3)$ matrix. The first three columns are given by an identity matrix

(A1) $${\left\{\nabla_{p}\Phi \left(p\right)\right\}}_{1:3}=I_{3\times 3},$$

and the j+3rd column is given by the gradient of the *j*’th basis function

(A2) $${\left\{\nabla_{p}\Phi \left(p\right)\right\}}_{j+3}=\nabla_{p}\phi_{j}\left(p\right)=\left[\begin{array}{c} \frac{\pi n_{j}\left(1\right)}{L_{u,1}-L_{l,1}}c_{1}s_{2}s_{3}\\ \frac{\pi n_{j}\left(2\right)}{L_{u,2}-L_{l,2}}s_{1}c_{2}s_{3}\\ \frac{\pi n_{j}\left(3\right)}{L_{u,3}-L_{l,3}}s_{1}s_{2}c_{3} \end{array}\right],$$

with $s_{d}$ and $c_{d}$ defined for the three spatial dimensions d=1,2,3 as

(A3a) $$\begin{array}{c} s_{d}=\sin\left(\pi n_{j}\left(d\right)\frac{\left(p_{d}-L_{l,d}\right)}{\left(L_{u,d}-L_{l,d}\right)}\right)\frac{1}{\sqrt{\frac{1}{2}\left(L_{u,d}-L_{l,d}\right)}}, \end{array}$$

(A3b) $$\begin{array}{c} c_{d}=\cos\left(\pi n_{j}\left(d\right)\frac{\left(p_{d}-L_{l,d}\right)}{\left(L_{u,d}-L_{l,d}\right)}\right)\frac{1}{\sqrt{\frac{1}{2}\left(L_{u,d}-L_{l,d}\right)}}. \end{array}$$

The Jacobians of the basis functions used in (29) is a $3\times 3\times (N_{m}+3)$ matrix. The first 3 entries along the third dimensions are all zero-matrices

(A4) $${\left\{\nabla_{\mathrm{pp}}\Phi \left(p\right)\right\}}_{1}={\left\{\nabla_{\mathrm{pp}}\Phi \left(p\right)\right\}}_{2}={\left\{\nabla_{\mathrm{pp}}\Phi \left(p\right)\right\}}_{3}=0_{3\times 3},$$

and the (j+3)rd entry along the third dimension is given by

(A5) $$\begin{array}{cc} \; & {\left\{\nabla_{\mathrm{pp}}\Phi \left(p\right)\right\}}_{j}=\nabla_{\mathrm{pp}}\phi_{j}\left(p\right)=\\ \; & \left[\begin{array}{cc} -{\left(\frac{\pi n_{j}\left(1\right)}{L_{u,1}-L_{l,1}}\right)}^{2}s_{1}s_{2}s_{3} & \left(\frac{\pi n_{j}\left(1\right)}{L_{u,1}-L_{l,1}}\right)\left(\frac{\pi n_{j}\left(2\right)}{L_{u,2}-L_{l,2}}\right)c_{1}c_{2}s_{3}\\ \left(\frac{\pi n_{j}\left(2\right)}{L_{u,2}-L_{l,2}}\right)\left(\frac{\pi n_{j}\left(1\right)}{L_{u,1}-L_{l,1}}\right)c_{1}c_{2}s_{3} & -{\left(\frac{\pi n_{j}\left(2\right)}{L_{u,2}-L_{l,2}}\right)}^{2}s_{1}s_{2}s_{3}\\ \left(\frac{\pi n_{j}\left(3\right)}{L_{u,3}-L_{l,3}}\right)\left(\frac{\pi n_{j}\left(1\right)}{L_{u,1}-L_{l,1}}\right)c_{1}s_{2}c_{3} & \left(\frac{\pi n_{j}\left(3\right)}{L_{u,3}-L_{l,3}}\right)\left(\frac{\pi n_{j}\left(2\right)}{L_{u,2}-L_{l,2}}\right)s_{1}c_{2}c_{3} \end{array}\right.\\ \; & \left.\cdots \begin{array}{c} \left(\frac{\pi n_{j}\left(1\right)}{L_{u,1}-L_{l,1}}\right)\left(\frac{\pi n_{j}\left(3\right)}{L_{u,3}-L_{l,3}}\right)c_{1}s_{2}c_{3}\\ \left(\frac{\pi n_{j}\left(2\right)}{L_{u,2}-L_{l,2}}\right)\left(\frac{\pi n_{j}\left(3\right)}{L_{u,3}-L_{l,3}}\right)s_{1}c_{2}c_{3}\\ -{\left(\frac{\pi n_{j}\left(3\right)}{L_{u,3}-L_{l,3}}\right)}^{2}s_{1}s_{2}s_{3} \end{array}\right]. \end{array}$$
